# Quadriplegia Following Prevertebral/Retropharyngeal Collection Secondary to Extrapulmonary Spinal Tuberculosis (Pott’s Disease)

**DOI:** 10.7759/cureus.62442

**Published:** 2024-06-15

**Authors:** Adnan Abdullah, Ehsan Ul Shakoor, Tasnuba Raisa Jamil, Anum Hina

**Affiliations:** 1 Acute Medicine, Queen's Hospital, Barking, Havering, and Redbridge University Hospitals NHS Trust, London, GBR; 2 Acute Frailty, King George Hospital, Barking, Havering, and Redbridge University Hospitals NHS Trust, London, GBR; 3 Internal Medicine, Sahiwal Medical College, Sahiwal, PAK

**Keywords:** rare tuberculosis involvement, pott’s spine, complicated retropharyngeal abscess, acute quadriplegia, extrapulmonary tb

## Abstract

Tuberculous retropharyngeal abscess, though rare, poses significant diagnostic and therapeutic challenges due to its atypical presentation. We present the case of a 51-year-old male with a history of drug abuse and inhalational burn injury presented with generalized weakness, sensory deficits, and neurological symptoms. Despite initial negative investigations, subsequent MRI and microbiological studies confirmed a rare case of tuberculous retropharyngeal abscess. The patient underwent urgent drainage and anti-tubercular therapy, experiencing complications such as *Candida* infection that required prolonged hospitalization and multidisciplinary care. This case underscores the importance of considering tuberculosis in differential diagnosis, especially in patients with unusual presentations and predisposing factors. It highlights the need for comprehensive evaluation, early intervention, and multidisciplinary management to prevent complications and improve outcomes. The case serves to raise awareness among clinicians about this uncommon presentation, emphasizing the need for a high index of suspicion in high-risk individuals and the importance of long-term follow-up and adherence to anti-tubercular therapy.

## Introduction

Tuberculous retropharyngeal abscess is an uncommon condition, but it can have serious consequences if not managed appropriately [1]. Tuberculosis (TB) primarily affects the lungs, but it can also manifest in other parts of the body, a condition known as extrapulmonary TB. Notable extrapulmonary TB sites include the lymphatic system, pleura, central nervous system (CNS), bones and joints (In decreasing frequency: lumbar, thoracic, and cervical vertebrae) and genitourinary system. Given its atypical presentation, diagnosing extrapulmonary TB can be challenging, necessitating a heightened level of suspicion. Typically, it arises as a complication of tuberculosis affecting the cervical spine. [2] Destruction of the vertebral spine by TB is also known as Pott's disease. [3]

TB is a bacterial infection caused by *Mycobacterium tuberculosis*. It normally spreads through inhalation of small droplets from the coughs and sneezes of infected persons, especially in poorly ventilated areas. In 2022, 1.3 million people lost their lives due to TB, including 167,000 individuals with HIV. Globally, TB ranks as the second most deadly infectious disease, surpassing both HIV/AIDS and COVID-19. In 2022, approximately 10.6 million people contracted TB globally. This included 5.8 million men, 3.5 million women, and 1.3 million children. TB affects people of all ages and is present in every country. The good news is that TB is both curable and preventable. [4] 

According to the TB incidence and reporting in England, in 2021, the incidence of TB was 7.8 cases per 100,000 population, which falls below the World Health Organization (WHO) threshold for a low-incidence country (defined as less than or equal to 10 cases per 100,000 population). TB in England disproportionately affected the most deprived populations. These vulnerable groups included individuals at risk of exclusion and other health disparities. [5] 

## Case presentation

A 51-year-old White British male was brought to the Accident and Emergency (A&E) by ambulance with a two-month history of generalized weakness and decreased sensation with numbness and tingling in all four limbs and torso. The patient mentioned that for the past few weeks, he had worsening symptoms and found it very difficult to mobilize, which was associated with occasional electric shock sensations going down the upper limbs to the neck. He denied any recent trauma to the head or neck. The patient had no fever, chest pain, shortness of breath, abdominal pain, diarrhoea, or urinary symptoms. 

He had a history of drug abuse, mainly opioids and cocaine. He was admitted in 2022 with epiglottitis, arytenoid oedema and airway narrowing secondary to inhalational burn injury (metal scourer from smoking cracked cocaine). He had CT neck and thorax on that setting which showed extensive edematous thickening of the supraglottic laryngeal airway and no obvious foreign body seen in the pharynx, larynx or the tracheobronchial tree. 

**Figure 1:**
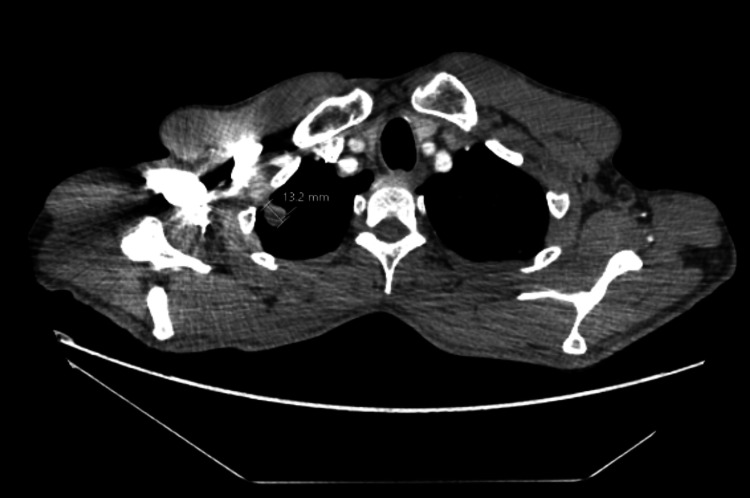
CT thorax with contrast (done six minths prior to current admission) showing a pulmonary nodule in the right upper lobe measuring in cross section 13.2 mm

He was admitted six weeks prior to the current presentation with abdominal pain and opioid withdrawal. He had raised inflammatory markers on that admission. CT thorax (Figure 1), abdomen, and pelvis were arranged to identify the source of infection which showed focal areas of branching centrilobular nodules seen in all the segments of bilateral upper lobes and the anterior-basal segment of the right lower lobe, likely infective aetiology. There were solid and ground glass nodules in the bilateral upper lobes and the right lower lobe. He was discharged with outpatient respiratory team follow-up. The outcome was to have an interval CT thorax after six months. 

On presentation, his Glasgow Coma Scale (GCS) was 15/15 and he was quite cachectic and unkempt. The pulse was regular with a good capillary refill, warm, and well-perfused. The chest was clear and heart sounds were normal with no added murmurs. On neurological examination, cranial nerves II-XII were intact. The tone was normal in all four limbs. There were no pronator drift and no cerebellar signs. Power was 4/5 in upper limbs proximally (4-/5 in distal muscle groups), 4+/5 in proximal muscle groups in lower limbs, and 4-/5 in distal muscle groups of lower limbs. Hyperreflexia was present in the knee jerk, biceps reflex, triceps reflex, and supinator reflex bilaterally. The ankle clonus presented bilaterally 20+ beats on each side. The patient had an ataxic gait on examination with decreased sensation in all four limbs, numbness and tingling on assessment, and normal sensation on the forehead. 

**Figure 2:**
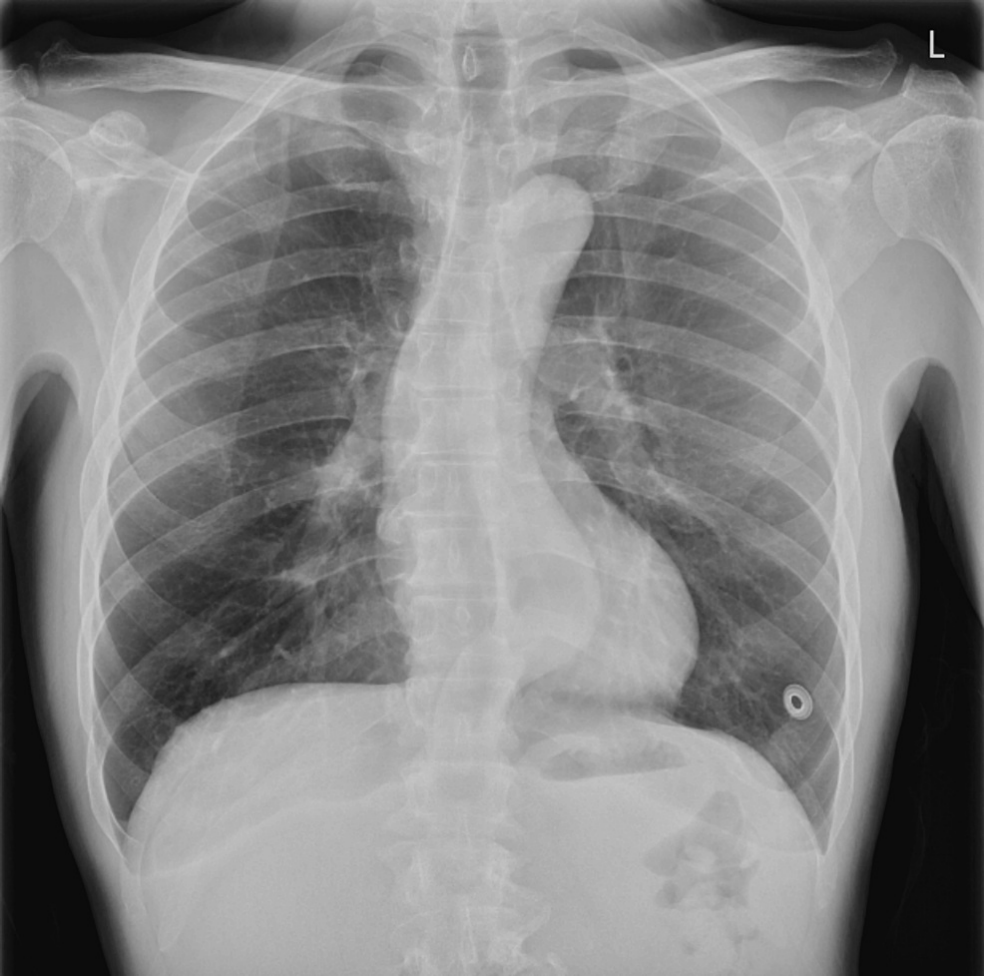
X-ray of chest at the time of admission showing both the lung fields equally inflated and clear. The hila, mediastinum, bony thorax, and pleura appear normal. The costo-phrenic and cardio-phrenic angles are clear.

Table 1 shows blood test results showing slightly elevated C-reactive protein (CRP) while the white blood cells were within the normal range. Viral markers were also negative. Chest X-ray appeared grossly normal (Figure 2).

**Table 1:** Laboratory results at the time of admission. These included routine blood test, liver and kidney function test, and inflammatory along with viral markers.

Parameters	Patient Values	Reference Range
Haemoglobin	131 g/L	133 – 173 g/L
White Blood Cells	9.5 10 *9/L	3.8 - 11.0 *9/L
Neutrophil	7.8 10*9/L	2.0 - 7.5 *9/L
Lymphocytes	1.0 10*9/L	1.5 - 4.0 *9/L
Prothrombin Time	10.8 s	9 – 13 s
Activated Partial Thromboplastin Time	25.1s	20 – 33 s
Sodium	141 mmol/l	133 – 146 mmol/l
Potassium	4.5 mmol/l	3.5 - 5.3 mmol/l
Urea	5.8 mmol/l	2.5 - 7.9 mmol/l
Creatinine	65 umol/l	60 – 104 umol/L
Total Bilirubin	5 umol/l	1-21 umol/L
Albumin	43 g/L	35-50 g/L
C-Reactive Protein	9 mg/L	<5 mg/L
Vitamin B12	466 ng/L	191 – 663 ng/L
Folate	4.5 ug/L	3.9 - 26.8 ug/L
HIV	Negative	
Hepatitis B	Negative	
Hepatitis C	Negative	

CT Head showed no evidence of an intracranial haemorrhage or major vessel territory infarction. Mild involutional changes proportionally to the age of the patient. Normal ventricular configuration with patent basal cisterns. No midline shift or hydrocephalus. Normal grey-white matter differentiation.

**Figure 3:**
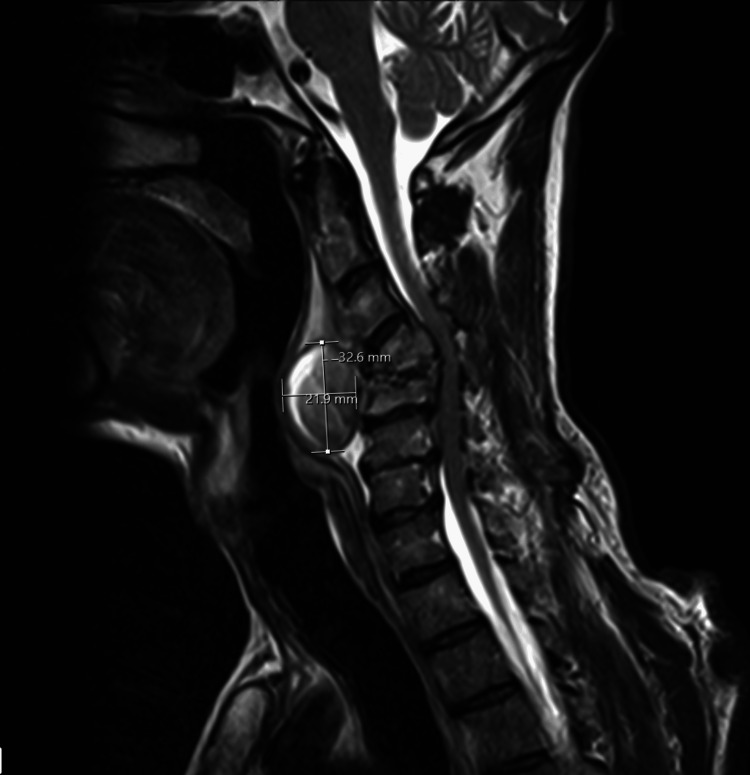
MRI spine with contrast with T2 sequence, done at the time of admission admission. There is a rim enhancing multiloculated collection measuring at least 5.5x3.5x2.5 cm in the prevertebral soft tissues in continuity with the C4-5 disc

MRI spine with contrast showed an abnormal signal in the C4-5 disc with enhancement with vertebral body end plate erosion, collapse, and an associated displacement and retropulsion of the vertebral bodies into the cervical canal. This resulted in spinal canal stenosis compression of the traversing cord and high short tau inversion recovery (STIR) malacic signal in the cord from C4 to C6-7 levels. There was a rim-enhancing multiloculated collection measuring at least 5.5x3.5x2.5 cm (about 0.98 inches) in the prevertebral soft tissues in continuity with the C4-5 disc. Figure 3 shows the MRI spine with contrast in the T2 sequence (done at admission) outlining the retropharyngeal abscess anterior to the cervical vertebrae. 

Given the findings, the patient was referred urgently to Neurosurgery and ENT. He was put on Vista® Collar (Aspen Medical Products, LLC, Irvine, California, United States) and started on IV dexamethasone to reduce oedema. The patient underwent transoral drainage of the prevertebral collection. Pus was collected and sent to Microbiology. He was treated with IV antibiotics including teicoplanin, metronidazole, and amikacin. After the procedure, the patient went to the intensive therapy unit (ITU) to maintain the airway. He had initially failed extubation due to a high secretion load and then was able to be successfully extubated.

Microbiology report showed that direct stain for acid alcohol fast bacilli (AAFB), as well as the TB polymerase chain reaction (PCR), were positive, and the region coding for the B-subunit of the RNA polymerase contained no mutations associated with rifampicin resistance.

The patient was then started on an anti-tubercular regimen. He went for recovery in one of the neurosurgical wards. While in the hospital, the patient developed a cough which was culture positive for *Candida albicans*. The patient continued to be on a hard collar while being reviewed by the Physiotherapy team. After treatment for the *Candida* infection, the patient made a good recovery.

**Figure 4:**
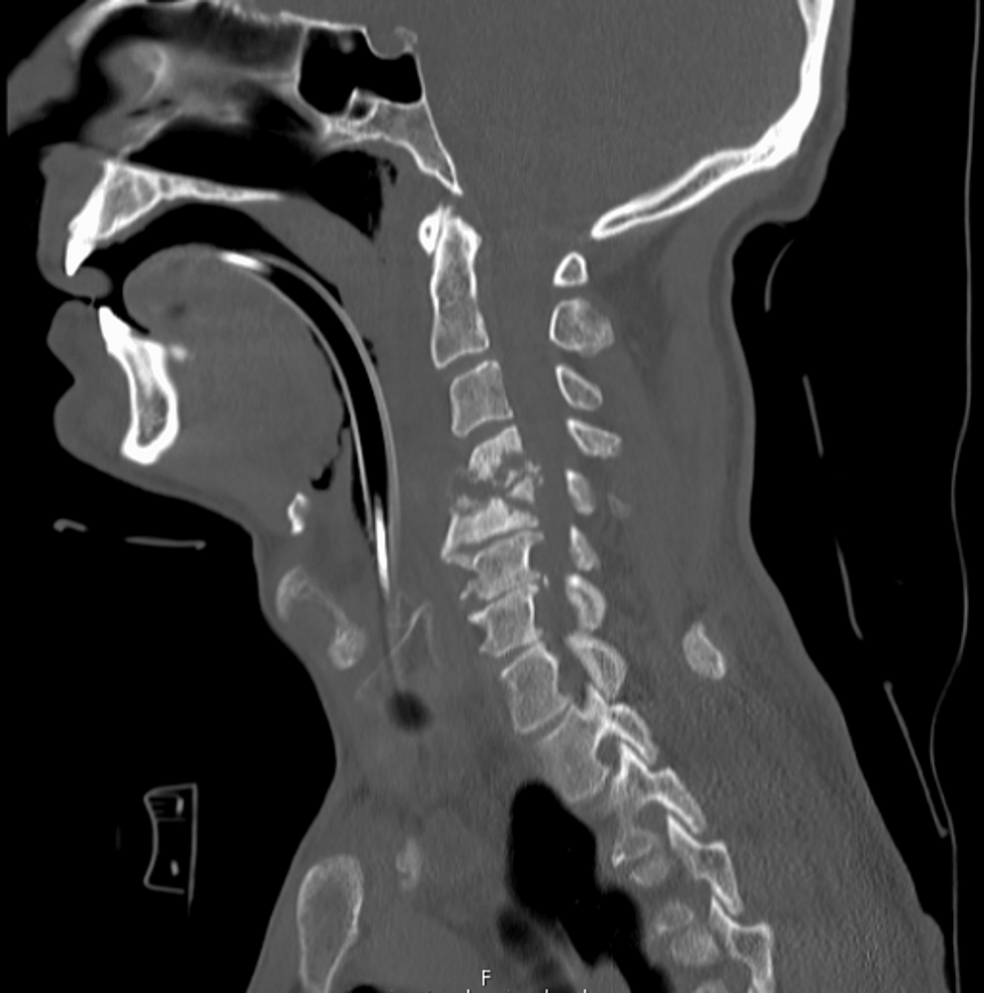
CT neck post drainage of the collection. The prevertebral collection has decreased significantly in size. There is small residual soft tissue thickening with some bone fragments within the prevertebral region. The thin rim of retropharyngeal oedema is seen extending from C2-C5. Bony destruction is seen involving the C4 and C5 vertebral bodies with mild retropulsion which extends into the spinal canal causing canal narrowing, which remained unchanged

Figure 4 shows a repeat CT scan of the neck after the transoral drainage.

The patient will be on long-duration anti-tubercular medication with rifampicin, isoniazid, pyrazinamide, and ethambutol for 12 months and will continue to be under follow-up from the TB team, Respiratory Medicine team, and Neurosurgery team. 

## Discussion

Tubercular retropharyngeal abscess is rarely reported in the Western world. Even fewer cases have been reported in the United Kingdom [1]. In the current case, there could have been predisposing factors that led to the presentation of retropharyngeal collection. In view of the bony destruction seen in the cervical spine, we can say that this finding is secondary to Pott's disease. Other reasons may include the inhalational burn injury back in 2022. The patient also has a history of recreational drug use with cocaine and heroin and probably comes across individuals having TB. Retropharyngeal abscess commonly occurs with traumatic neck injury in adults. Inhalational burn injury acts as a source of trauma making the patient more susceptible. Furthermore, the patient also had poor socioeconomic status. An early high index of suspicion is necessary, especially in patients with lung findings from the scan. TB should be excluded in such patients before moving to further investigations. Early diagnosis and interventions are crucial to prevent further complications such as the development of retropharyngeal abscess. 

Currently, there are several tests to diagnose latent and active TB. For latent TB, interferon-gamma release assay (IGRA) is readily available across laboratories in the united Kingdom. Tuberculin skin testing can be another method [6]. Neither test can diagnose or exclude active TB and can be falsely negative in 20-25% of cases. For active TB, sputum culture is thought to be the gold standard with isolation of the *Mycobacterium tuberculosis *bacteria. It takes around one to three weeks for culture to grow in liquid media and around four to eight weeks in solid media [7]. As a result, nucleic acid amplification testing is gaining popularity by directly detecting the DNA or RNA in sputum. It provides rapid diagnosis within eight hours [8]. 

## Conclusions

This case highlights the uncommon presentation of tuberculous retropharyngeal abscess in a patient with a history of drug abuse. It’s worth noting that a retropharyngeal abscess due to spinal TB, although uncommon, is a known presentation of the disease. Certain injuries in the neck and mouth, such as those caused by inhalation, can increase the risk of developing a retropharyngeal abscess. The management of a tubercular retropharyngeal abscess involves early intervention, which includes draining the abscess, providing support, and initiating anti-TB medication. 

The case underscores the importance of maintaining a high index of suspicion for TB, especially in patients with a history of drug abuse and those presenting with atypical symptoms. Early diagnosis and intervention, including the initiation of an anti-tubercular regimen, are crucial in managing such cases and preventing further complications. This case also emphasizes the need for a comprehensive and multidisciplinary approach in the management of such complex cases. 
